# Relationships between Community Festival Participation, Social Capital, and Subjective Well-Being in a Cross-Cultural Context

**DOI:** 10.3390/healthcare11162361

**Published:** 2023-08-21

**Authors:** Young-joo Ahn, Eunice Minjoo Kang, Kiattipoom Kiatkawsin, Seweryn Zielinski

**Affiliations:** 1 Department of Hospitality and Tourism Management, Sejong University, Seoul 05006, Republic of Korea; yjahn@sejong.ac.kr (Y.-j.A.); emk3620@gmail.com (E.M.K.); 2 Business Communication and Design, Singapore Institute of Technology, 10 Dover Drive, Singapore 138683, Singapore; kiatkawsin@gmail.com

**Keywords:** subjective well-being, happiness, life satisfaction, community engagement, community festival, cross-cultural studies

## Abstract

This study explores the relationships among social capital, community festival participation, and subjective well-being (SWB). It examines the mediating role of festival participation between social capital and SWB. The dataset Social Well-being Survey in Asia from the Philippines and Thailand was collected using nationwide surveys in 2016. The total number of respondents was 1057 in the Philippines and 982 in Thailand. The results affirm several determinants related to SWB, which is composed of happiness and overall life satisfaction. The models show significant relationships among festival participation, social capital, and SWB. The results indicate strong associations among social capital with family and relatives, festival participation, and SWB. The interaction effects between the two countries are included. Structural and cognitive social capital with kinship groups were important determinants in facilitating festival participation, and positively associated with SWB. Moreover, the results identify the mediating effect of festival participation between social capital with family and relatives and SWB. The results can provide similarities and differences in the relationships among social capital and kinship groups, community festival participation, and SWB between the Philippines and Thailand. This study offers important empirical evidence of a cross-cultural study in the context of the Philippines and Thailand.

## 1. Introduction

Economic growth has been a priority in many countries and increasing income is one of the important goals [1]. However, inequality and social problems have been increasing and have not been fully solved [2]. The indicator of economic growth, Gross Domestic Product (GDP), does not guarantee the improvement of individuals’ subjective well-being (SWB) and additional indicators have been sought for understanding and evaluating various regions around the world “beyond” GDP [1,3,4].

The World Happiness Report published in 2022 indicated additional indicators beyond GDP and assessed the level of positive and negative emotions, happiness, and SWB [3]. According to the World Happiness report published in 2019 and 2022, the top three countries regarding happiness were Finland, Denmark and Norway. Individuals were asked to fill out items related to their perceived happiness on a Likert scale from 0 to 10 points, and Thailand (M = 6.008) for 2016 to 2018 ranked 52nd out of 156 countries and the Philippines (M = 5.631) ranked 60th. Thailand (M = 5.891) for 2019 to 2021 ranked 61st out of 146 countries and the Philippines (M = 5.904) ranked 60th [3,5]. Nationality is a critical factor in exploring similarities and differences in cross-cultural contexts and is considered in different fields, such as tourism [6], happiness studies [7], and community studies [8,9]. The Philippines and Thailand are important countries in Southeast Asia, and many institutes and NGOs in these countries have cooperated in cross-cultural studies to provide a deeper understanding of their inter- and intra-regional characteristics and build effective and efficient strategies for better communities [8]. Moreover, the data availability of nation-wide data in cross-cultural contexts is a crucial element in the generation of important implications by scholars [10]. The cultural comparisons of cultural values and SWB among different countries have increased our understanding of how people perceive and evaluate their lives [1].

Previous research has investigated the indicators for better societies around the world and linked social issues with important concepts such as social capital, collaborative actions, and SWB [11]. One of the indicators strongly associated with SWB is social capital [12]. Individuals with more and good-quality social relationships show more positive emotions and behaviors [13,14]. They shape, develop, or increase social relationships, wherein social capital is able to conceptualize and operationalize social networks and social bonds [14]. People build trust with kinship groups and other social groups [14]. Although social capital is complex, social relationships in family and kinship relationships are likely to be associated with trust-based relationships and the enhancement of positive consequences. Previous studies have explored the determinants of subjective wellbeing and affirmed the positive association between social capital and SWB [12,15]. Collective actions are facilitated by social networks and trustful social ties [14]. Collective actions in communities can be local festivals in communities [16,17,18,19,20,21]. However, the extant research on social capital, festival participation, and SWB has rarely been explored by using the cross-cultural data of the Philippines and Thailand.

Therefore, this study aims to explore the relationships among social capital, community festival participation, and SWB. It aims to examine the mediating role of festival participation between social capital and SWB. The results can contribute to providing insights into the similarities and differences in the relationships among social capital, community festival participation, and SWB between two countries, namely the Philippines and Thailand.

## 2. Literature Review

### 2.1. Social Capital

Social capital refers to “features of social organization, such as networks, norms, and trust that facilitate coordination and cooperation for mutual benefit [14], pp. 35–36.” Previous studies have indicated the complexity of social capital [13]. Many scholars have elaborated the meaning and the multiple concepts of social capital [13,22,23]. Social capital cannot be described simply because it can be newly connected and bonded. However, it can be ceased [13]. The important components of social capital are bridging social capital (i.e., social relations with various social identity groups) and bonding social capital (i.e., strong social ties among social groups with similar characteristics) [13,22]. Structural social capital refers to observable social relationships. However, cognitive social capital refers to social groups sharing similar values and norms of reciprocity [24]. Social capital is positively linked to increasing opportunities for exchange, such as sharing knowledge, resources, values, social norms, trust, and reciprocity [25,26,27].

Previous studies have exerted efforts to identify the properties of social capital and operationalize social capital based on social networks, social structure, interaction frequency, the quality of social relationships, and trust in various social groups [13,22,23]. However, there is no consensus with regard to social capital indicators. Social capital is assessed based on the quantity and quality of social relationships [28,29]. Previous research has indicated the positive effects of social capital. For example, individuals with a high social capital are likely to report positive mental health, low depression, and a low rate of alcohol abuse [24]. However, individuals lacking social capital are likely to perceive social isolation and feelings of loneliness and show a high mortality rate and suicide rate [29,30].

### 2.2. Social Capital and SWB

Previous studies have demonstrated that social capital is one of the essential predictors of SWB [12,15,29,30,31,32,33,34,35]. SWB has been operationalized according to the individuals’ overall life satisfaction and positive and negative emotions such as happiness, stress and depression [36]. The extant literature on social capital and SWB has identified that cognitive social capital exhibits a stronger association with SWB than structural social capital [12]. The core components of social capital such as social network size, trust, and interaction with social ties are positively associated with happiness and life satisfaction [33,34]. Family size, informal social ties, and the frequency of interaction are identified as important components of social capital, which is associated with SWB [35].

### 2.3. Festival Participation, Social Capital, and Subjective Well-Being

Community engagement refers to the extent to which residents participate in activities in their community [37]. Local festivals can be viewed as collective actions and celebrations among relevant actors, including community residents. The benefits of festivals have been illustrated well [38,39]. Various types of festivals can provide economic boosts, as well as social cultural benefits such as social engagement, self-congruence, the sense of cohesiveness, commitment, and pride [40,41]. Festival participants can relax from daily life, feel refreshment, enjoyment and a meaning of life in a festive celebration atmosphere at an individual level [42,43]. Spending time with family members and important social networks such as friends and neighbors is important during festivals [44].

Family and close friends are likely to enjoy gatherings at festival venues. Festival participants sometimes interact with other festival participants, but they avoid interactions with strangers [45]. Japson, Stadler, and Spencer [46] identified that family bonding, connections with family members and positive memories, and individuals’ quality of life were the core categories of their study, which used a qualitative approach.

### 2.4. Control Variables

Previous studies on festival participation, social capital, and SWB have demonstrated the effects of demographic and socio-economic characteristics and examined numerous models controlling for demographic and socio-economic characteristics such as age, gender, marital status, income, education level, employment status, and residential areas [28,31,35,47,48]. Regarding demographic characteristics, age shows a U-shape effect on SWB [12,28]. Older adults who have rich resources, support from family members, and participate in social activities show a high level of happiness and life satisfaction [49]. Gender and marital status are also important demographic characteristics in SWB studies [48,50]. Previous studies have not obtained consistent results in various research contexts. However, the results have indicated that females show higher SWB than males. The characteristics of a residential area (e.g., urban, rural) can be an influential determinant of residents’ SWB [48]. Previous studies have found that individuals with a higher level of education, who are currently working, and who have a higher income are more likely to take vacations and show high SWB [42,48]. Cross-cultural studies use residence areas, socio-cultural factors, and ethnicities to examine the differences in and critical determinants of SWB [51]. This study considered demographic and socio-economic characteristics as control variables in the proposed models based on previous studies.

### 2.5. Hypotheses

#### 2.5.1. Social Capital and Festival Participation

Social structures and social networks with family, friends, and organizations facilitate further interaction and build trustful relationships. Moreover, individuals with a larger social network, more social connections, and higher social trust tend to join leisure activities, promote collective action, and engage in political campaigns [14,28,30,31]. The accumulation of social capital leads to the high probability of being involved in social activities, volunteering, and religious activities [30]. Individuals can also build social networks through participation in leisure and sport in communities [52]. The development of social capital deepens shared values and trust, and increases the extent of individuals’ commitment to social ties and group cooperation [27]. Shared identity and congruence facilitate engagement in group collaborations in organizations, which is reflected in the volume of social capital accumulated and increases in organizational commitment and the exchange of knowledge [27,31,41].

A handful of studies have examined the relationship between social capital and festival participation [19,20,21,45,53]. The complexity of social capital has conceptually and empirically explored how individuals create and enhance social relationships before, during, and after events and festivals [21]. Visitors may be motivated to participate in events and festivals due to existing social capital, social identity, and personal values [19,45,54]. Trust-based social ties also increase festival participation [45,55]. Therefore, we proposed the following:

**H1.**  *Social capital is positively associated with festival participation when controlling for demographic and socio-economic characteristics.*

#### 2.5.2. Social Capital, Festival Participation, and SWB

Individuals who indicate that they have high-quality social relationships with close social ties are more likely to report a high SWB [12]. The social network type is an important predictor of SWB [35]. This study focuses on family interaction and trustful relationships with family and relatives, and examines the relationship between social ties with family and relatives and SWB [24]. In a different cultural context such as Thailand, social capital is also identified as an important predictor of happiness, low levels of anxiety, and participation in public hearings and activities with mutual benefits [56].

Community engagement, such as community festival participation, is one of the important determinants of overall life satisfaction [12]. Festival experience is positively associated with improvements in individuals’ quality of life and psychological well-being [40,44,50,57]. For example, Wood and Moss [44] attempted to capture the live emotional states of music festival participants and found a strong sense of belonging to the festival. Therefore, we proposed the following:

**H2.**  *Social capital is positively associated with SWB when controlling for demographic and socio-economic characteristics.*

**H3.**  *Festival participation is positively associated with SWB when controlling for demographic and socio-economic characteristics.*

#### 2.5.3. The Mediating Effect of Festival Participation

Previous research [28] has identified the relationships between festival participation, informal social ties, and SWB. It has demonstrated the mediating role of festival participation between close social groups, as well as SWB, among Koreans. Chung [41] examined the effect of the perceived benefits of seeking an ethnic sport event on SWB, visitor satisfaction, and organizational commitment. Mesana and de Guzman [43] recently examined festival happiness and the positive consequences of festival participation among Filipino participants by using a qualitative research approach. They found the social-cultural benefits of local festivals and affirmed the links among festival experience, individuals’ social–cultural quality of life and residents’ overall life satisfaction. Lei and colleagues [35] indicated that kinship relationships, social interactions, the social group size, and the hours of social activity participation can be important predictors for SWB. Therefore, we proposed the following:

**H4.**  *Festival participation shows a mediating effect for the relationship between social capital and SWB when controlling for demographic and socio-economic characteristics.*

## 3. Methods

### 3.1. Data Source

The data were shared by KOSSDA [10], and were collected through the ‘Social Well-being Survey in Asia (SoWSA)’; this project was led by Senshu University in Tokyo, Japan. SoWSA aims to evaluate SWB in East and Southeast Asian countries. A standardized survey instrument was developed by the Center for Social Well-being Studies at Senshu University and then was translated into national languages. The datasets of two countries (the Philippines and Thailand) were collected using the nationwide surveys in 2016. The data from the Philippines were collected by using face-to-face interviews with participants from June to December 2016 via two-stage and quota sampling. The fieldwork institution was Ateneo de Manila University in the Philippines. The data in Thailand were collected by using face-to-face interviews with participants from October to December 2016 via proportionate quota sampling. The fieldwork institution was Chulalongkorn University Social Research Institute in Thailand.

The collected dataset was cleaned and distributed by KOSSDA, located in Seoul National University. There were a total of 1200 respondents in the data from the Philippines and a total of 1114 respondents in the data from Thailand. After removing unusable observations with missing data and selecting an age range between 20 and 69, the total number of respondents available for the final analysis was 1057 in the Philippines and 982 in Thailand.

### 3.2. Measures

Subjective wellbeing was assessed using two dependent variables, namely happiness and overall life satisfaction. Happiness included one measurement item, ‘How happy are you currently?’ Overall life satisfaction included one measurement item, ‘How satisfied are you currently with your life?’ Two items were measured by using an 11-point Likert scale from 0 = “very unhappy” to 10 = “very happy”, and from 0 = “very unsatisfied” to 10 = “very satisfied.”

Social capital was measured using two aspects, namely structural social capital and cognitive social capital. Structural social capital was measured by using the level of interaction with social ties, such as family and relatives, friends, and neighbors, and the level of interaction ratio with neighbors. The interaction with close social ties was assessed according to the frequency of interaction with social ties and used a five-point Likert scale. Cognitive social capital was assessed according to trust in social ties. The items of the survey were measured to identify the level of trust and various informal social ties (e.g., family and close relatives, friends, neighbors). The measurement items were assessed by using a five-point Likert scale from (1) “I cannot trust at all” to (5) “I can trust a lot” [10].

The measurement items about the interaction with and trust in various social ties were available from KOSSDA [10], and the specific measurement items can be found from KOSSDA [10,28]. With regard to festival participation, the survey item of festival participation was measured using a five-point Likert scale, from (1) “never attend” to (5) “I usually attend”. With regard to the control variables, demographic characteristics were added in the models; these included age, gender, marital status, income, and residential area. The individual characteristics were identified as important variables associated with SWB. Age was used as a continuous variable. Gender and marital status were dummy coded. Female was used as reference. Education was divided into five categories from (1) lower secondary to (5) masters or doctorate degree. Regarding regions, this item was divided into residence area (0: living in rural area, 1: living in urban area) and country (0: Thailand, 1: the Philippines,). Employment state (0: not working, 1: working) was dummy coded.

### 3.3. Analysis

First, the descriptive analysis was calculated to present the descriptive results concerning the respondents’ demographic characteristics and the level of SWB (i.e., happiness and overall life satisfaction) based on a total of 28 regions in two countries. Second, a Pearson correlation analysis was computed. Finally, an ordinal logistic regression analysis was calculated to test the relationship between the groups of social capital, festival participation, happiness, and overall life satisfaction by controlling for the demographic and socio-economic characteristics of the respondents. The recommended threshold values were used based on the previous literature [58]. A mediation analysis was also used to identify the mediating effect of festival participation between social capital and two SWB items by controlling for demographic and socio-economic characteristics [59,60,61]. This study used STATA 17 for statistical analysis.

## 4. Results

### 4.1. Coding Information of the Data and the Descriptive Results

Table 1 provides the coding information of the data and the descriptive results. The two countries were separately presented. Regarding the coding information, the age ranged from 20 and 69 and was used as a continuous variable; age was centered in the models.

Gender was dummy coded (male = 1, female = 0). Marital status was dummy coded (married = 1, others = 0). Education included five categories from 1 to 5 and measured the level of education. Residence area was dummy coded (urban = 1, rural = 0). Employment was dummy coded (working = 1, not working = 0). Happiness was categorized into three groups (low = 0–4 points; medium = 5–7 points; high = 8–10 points). Overall life satisfaction was categorized into three groups (low = 0–4 points; medium = 5–7 points; high = 8–10 points). Interaction with social groups and the ratio of interaction with neighbors were measured from 1 to 5. Trust in social groups was measured from 1 to 5. Finally, festival participation was measured from 1 to 5. The descriptive information is presented below in Table 1. All social capital groups, namely family, relatives, friends, and neighbors, were considered in the initial stage. However, other social groups did not show significant relationships with SWB. Consequently, this study used social capital with family and relatives between the two countries.

Table 1 provides the demographic results for the respondents of the two countries.

The mean age of the respondents in the Philippines was 42, while that of the respondents in Thailand was 45, but the age groups vary. Approximately 50.52% of the respondents in the Philippines were female, while 53.26% of the respondents in Thailand were female. Approximately 73.13% of the respondents in the Philippines were married, while 63.24% of the respondents in Thailand were married. Approximately 11.35% of the respondents in the Philippines reported had Bachelor’s degrees, while 21.18% of the respondents in Thailand reported that they had Bachelors’ degrees. Approximately 65.37% of the respondents in the Philippines reported that they work, while 82.99% of the respondents in Thailand reported that they worked.

### 4.2. Correaltion Matrix

The Pearson correlation matrix of eight variables is presented in Table 2. The correlation values range from 0.031 to 0.651. The relationship between interaction with family and relatives and trust in friends is not statistically significant and shows a negligible value in the correlation matrix. Most relationships show weak and moderate correlation values and are statistically significant at *p* < 0.05 level.

### 4.3. SWB According to Regions in Two Countries

**Figure 1:**
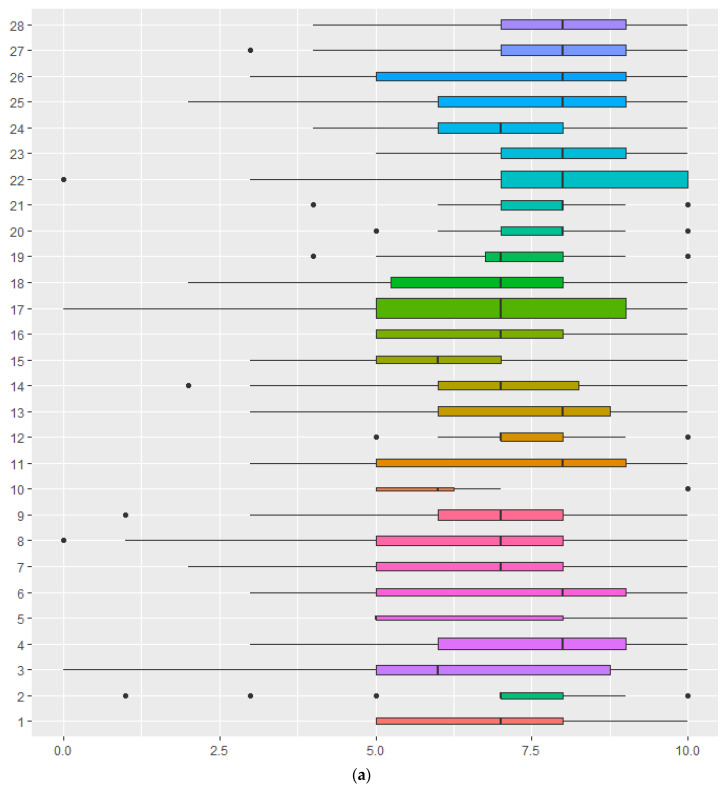

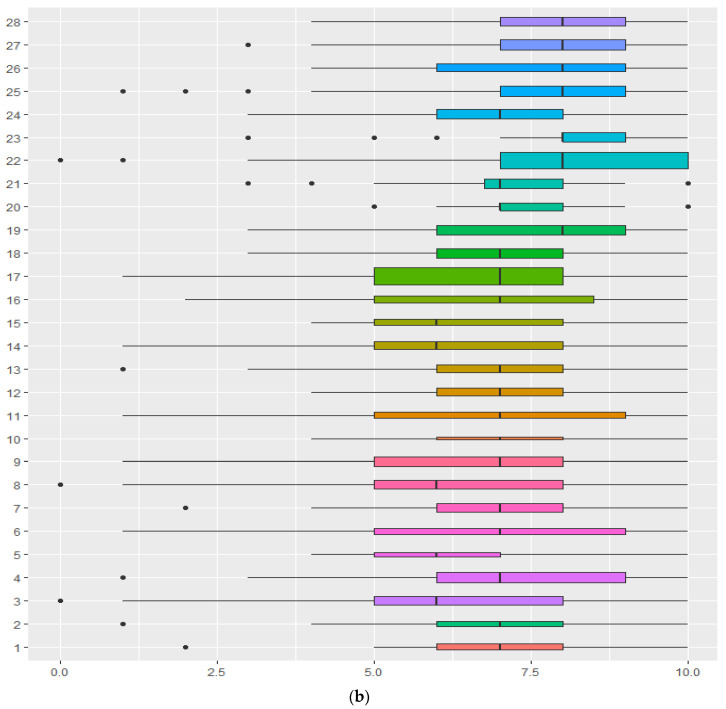
The box plots of subjective well-being according to the regions of the two countries. (**a**) Happiness and region. (**b**) Overall life satisfaction and region. Note 1: Y axis: Region = 1–17 (The Philippines) and 18–28 (Thailand); X axis: 0–10 points of happiness in Figure 1a and overall life satisfaction in Figure 1b. Note 2: A total of 28 regions across the two countries; ranks of SWB based on regions in parentheses. Note 3: Black dots are individual data points that are shown outside the box plots.

Figure 1a,b shows the box plots of happiness and overall life satisfaction according to a total of 28 regions across the two countries. Information of X axis and Y axis is presented in Table 3. A total of 17 regions in the Philippines showed various levels of happiness and life satisfaction, while a total of 11 regions in Thailand showed similar patterns of happiness and life satisfaction at the municipal level. The regions that showed higher levels of happiness included Cavite (M = 7.372), Zamboanga del Sur (M = 7.317) and Camarines Sur (M = 7.306), and the regions that showed lower levels of happiness included Leyte (M = 6.250), Mindoro (M = 6.294), and Agusan del Norte (M = 6.364). The top three regions with a higher overall life satisfaction were Misamis Oriental (M = 7.31), Cavite (M = 7.279), and Davao del Sur (M = 7.138), while the three regions that showed a lower overall life satisfaction in the Philippines were Bulacan (M = 6.157), Mindoro (M = 6.529), and Negros Occidental (M = 6.313).

The levels of happiness and overall life satisfaction showed similar patterns in Thailand. The three regions in Thailand that showed high happiness and life satisfaction were Nakornpanom (M = 8.186, M = 8.233), Nakorn Sri Thammarat (M = 7.920, M = 7.632), and Ubonratchathani (M = 7.884, M = 7.786), while the three regions that showed a relatively lower level of happiness and life satisfaction were Bangkok (M = 6.849, M = 6.977), Chiang Mai (M = 7.299, M = 7.195), and Kanjanaburi (M = 7.358, M = 7.302).

### 4.4. Ordinal Logistic Regression Model

Ordinal logistic regression models, from Models 1 to 6, were performed after conducting several analyses. Other informal social groups such as friends and neighbors were not included in the models because these variables were not statistically significant in the models. As a result, this study focuses on the kinship relationships (i.e., family and relatives) of the two countries. Models 1 to 2 in Table 4 used control variables and social capital (i.e., interaction with family and relatives and trust on family) to estimate the association with festival participation. Models 3 to 6 comprised control variables, festival participation, and social capital, and the interaction between festival participation, social capital, and the two countries (i.e., Thailand and Philippines). Models 3 to 6 tested the relationships between the variables added in the model and subjective well-being. However, social capital regarding other social groups such as friends and neighbors was excluded in the final models because of its insignificance.

Models 1 and 2 showed that two demographic variables (i.e., marital status and education level) were significant. Married and individuals reporting a lower level of education status were more likely to attend a festival. Thai respondents living in rural areas were likely to show higher festival participation. As reported in Model 2, individuals with strong social capital with their family and relatives showed a 1.117 times higher trust in family and relatives and a 1.273 times higher interaction with family and relatives than those who reported weak social capital with family and relatives.

Models 3–4 in Table 5 comprised control variables, festival participation, social capital (i.e., trust on family and interaction with family and relatives), and interaction with two populations (i.e., Thais vs. Filipinos). As noted in the coding information, happiness was categorized into three groups (i.e., low, medium, and high groups of happiness). The older group in Model 3 reported a higher level of happiness than the younger group. When the other predictors were held constant, the odds ratio of festival participation between the given level (male) and the reference level (female) was 2.41 times lower (*p* < 0.05). Those who reported being married (OR = 3.22, *p* < 0.01), having higher education levels (OR = 5.60, *p* < 0.001), and currently working (OR = 2.29, *p* < 0.05) were likely to be happier than the opposite. Those who reported participating in festivals were likely to have a 3.34 times higher level of. With regard to social capital, those who reported trusting family and relatives were likely to be 2.26 times happier than those who reported lower trustful relationships with family and relatives. Those who reported having a closer interaction with family and relatives were likely to be 5.68 times happier than those who reported lower levels of interaction with family and relatives. The results of Model 4 added the interaction among festival, social capital, and the two countries (i.e., Thailand vs. the Philippines). Filipinos with more trustful relationships with family and relatives were less likely to report a higher level of happiness than Thais (OR = 0.805, *p* < 0.05).

Models 5 and 6 in Table 6 included the control variables of demographic and socio-economic variables, festival participation, social capital (i.e., trust in family and interaction with family and relatives), and interaction with two populations (i.e., Thais vs. Filipinos). The dependent variable was overall life satisfaction, which was categorized into three groups (i.e., low, medium, and high groups). The older age group in Model 5 reported a higher level of overall life satisfaction than the younger age group (OR = 1.001, *p* < 0.01). The odds ratio of festival participation between the given level (male) and the reference level (female) was 0.778 (*p* < 0.01), and female respondents showed a higher level of overall life satisfaction than males. Those who reported being married (OR = 1.402, *p* < 0.01) and having higher education levels (OR = 1.228, *p* < 0.01) were likely to show a higher level of life satisfaction than the opposite. Regarding social capital, those who reported trusting in family and relatives were likely to show a 1.159 times higher level of life satisfaction than those who reported having fewer trustful relationships with family and relatives. Moreover, those who reported having closer interactions with family and relatives were likely to show a 1.290 times higher level of life satisfaction than those who reported having lower levels of interaction with family and relatives.

The results of Model 6 were similar to those of Model 4 after adding the interaction among festival, social capital, and the two populations (i.e., Thais vs. Filipinos). Filipinos with festival participation were less likely to report a higher level of overall life satisfaction than Thais (OR = 0.755, *p* < 0.001). Regarding interaction with family and relatives, Filipinos were less likely to report a higher level of overall life satisfaction than Thais (OR = 0.818, *p* < 0.05). Social capital regarding trust in family and relatives did not show any statistical significance.

### 4.5. The Mediating Effect of Festival Participation

The mediating role of festival participation was identified by using the KHB method [59,60,61]. The direct, indirect, and total effects are presented in Table 7. Festival participation showed a significant relationship between the two social capital variables and happiness, and the results indicated the mediating effect of festival participation as controlling for demographic and socio-economic variables in Models 3–6. Table 7 shows the statistically significant mediating effect of festival participation among trust in family and relatives, interaction with family and relatives, and happiness. Approximately 8.15% of the total effect was due to festival participation. Interaction with family and relatives also showed that approximately 7.32% of the total effect was due to the mediation. However, the mediating effect of festival participation among the two social capital variables and overall life satisfaction was insignificant.

## 5. Discussion

### 5.1. Theoretical and Practical Implications

The results indicate several theoretical implications. First, the results affirm several determinants of happiness and overall life satisfaction. The models show significant relationships among social capital, festival participation, and SWB among individuals in the Philippines and Thailand. Consistent with previous research [28,32], structural and cognitive social capital and festival participation enhance SWB. Social interaction and trustful relationships with family and relatives may increase resources and opportunities to participate in community festivals. Moreover, the results show a statistically significant association between social capital with kinship groups and SWB among individuals in the Philippines and Thailand. The interaction effects of the two countries also indicate that festival participation and social capital with family and relatives show statistically significant results in positive association with SWB. This study highlights the strong associations among social capital with family and relatives, festival participation, and SWB in the models in Table 4, Table 5 and Table 6. Trust in and interaction with family and relatives were found to be important determinants in facilitating festival participation, as shown in Table 4 (H1: supported). Moreover, social capital with family and relatives is positively associated with SWB in Table 5 and Table 6 (H2: supported).

Second, the results indicate strong associations between festival participation and SWB (H3: supported). The interaction effects between the two countries for identifying differences between them are included in Models 4 and 6. The results indicate that older, female, married, and highly educated respondents are likely to present a higher level of SWB. Inconsistent with Ivlevs [48], residents in rural areas show an insignificant association with SWB. The results also reveal the interaction effect of nationality and reveal the differences between Thais and Filipinos. The results obtained from the dataset of this study indicate that Thai respondents are likely to show higher SWB than Filipino respondents. Akter et al. [8] indicated that female Filipinos have high responsibilities in farming and workloads and have relatively less time for leisure and relaxation than those in other Southeast Asian countries.

Finally, this study demonstrates the effect of festival participation. Thai respondents who are married, less educated, and living in rural areas are more likely to attend community festivals. Those with higher social capital with family and relatives are likely to attend festivals. Moreover, the results identify the mediating effect of festival participation between social capital with family and relatives and happiness. Individuals who participate in community festivals with kinship groups are likely to enjoy festive and entertaining atmospheres and feel happiness. However, the results did not find a mediating effect between social capital and overall life satisfaction on festival participation (H4: partially supported). In this study, the results did not show a mediating effect between festival participation and life satisfaction. The results may indicate that individuals’ life satisfaction is connected to contentment with various aspects of their lives, as indicated by Chen, Lehto, and Cai [42]. Further studies need to explore the relationships among social capital with various social groups, festival participation, and SWB.

This study provides important implications for practitioners. First, social capital with family and relatives positively affects SWB. Trustful relationships with family and relatives and interaction with them are linked to happiness and overall life satisfaction.

Growiec and Growiec [32] determined that kinship relationships (e.g., family members and relatives) are likely to develop social capital, which is strongly linked to SWB. Practitioners and governments can create community programs and various types of community festivals for family gatherings.

Second, festival participation is associated with SWB. Kinship groups can enjoy a festive atmosphere and attend festivals that may deepen family cohesion. Moreover, community festivals must provide various festival activities and programs that community members can participate in and gain entertaining and memorable experiences with kinship groups. Collective memories during festivals with family and relatives could lead to happiness. Mesana and de Guzman [43] indicated that practitioners and governments should develop festivals that can maintain ethnic heritage and roots, and increase the quality of festivals and manage the legacy of community festivals with relevant stakeholders in communities.

Finally, this study highlights the cross-cultural comparison of the results controlling for demographic and socio-economic characteristics. Moreover, as previously pointed out by Helliwell and Putnam [12], individuals with lower income and educational status show low SWB. Disadvantaged and vulnerable groups may have family conflicts or fewer resources than the opposite groups. Practitioners and governments need to develop intervention programs that help disadvantaged people use resources and get involved in activities and organizations in communities for building social capital, which could facilitate active community engagement and SWB. The results can provide insightful information for achieving sustainable development goals (SDGs) and building proactive plans for subjective well-being and sustainable communities.

### 5.2. Limitations and Suggestions for Future Studies

This study used nationwide and cross-cultural data. However, this study has some limitations. First, the results should not be generalized. This study is a cross-sectional study. Further research needs to replicate the proposed model and verify the findings. It also needs to conduct more cross-cultural studies that help to identify similarities and differences across various countries. Second, this study focused on kinship relationships and did not discuss the effects of various formal and informal social groups, such as friends, neighbors, colleagues, organizations, and governments in the proposed models. Moreover, the measurement of social capital needs to be developed. Future studies must examine the effect of social groups on SWB. Third, the control variables of this study used important demographic characteristics and socio-economic characteristics. Critical determinants such as religious and cultural behaviors may be considered. Fourth, this study uses a single measurement item of festival participation. This study cannot deeply explore the respondents’ festival experience and the role of festival participation in social capital and SWB. Future research should include specific festival experiences, different types of festivals, and specific information regarding festivals. Finally, the results cannot reflect changes in SWB before and after the festival experience. Multiple data collections over time or other methodologies, such as qualitative research approaches, will provide an insightful understanding of the effects of the festival experience.

## 6. Conclusions

This study examines the relationship among social capital, festival participation, and SWB. Moreover, it presents the decomposition effect of festival participation between social capital and SWB (i.e., happiness and overall life satisfaction). The results highlight the positive association among social capital with kinship groups, festival participation, and SWB. Moreover, the mediating effect and the interaction effects are found. This study provides important empirical evidence of a cross-cultural study in the context of the Philippines and Thailand. In addition, it provides a deeper understanding of the relationships among social capital with kinship groups, community engagement, such as community festival participation, and SWB by controlling for demographic and socioeconomic characteristics. Further studies should continue measuring SWB among different countries to increase the SWB of all individuals, build sustainable communities, and achieve the sustainable development goals.

## Figures and Tables

**Table 1:** Descriptive statistics for the sample.

	Philippines	Thailand	
Data Sample	*n* = 1057	*n* = 982	
Variable	*n* (%), Mean (SD)	*n* (%), Mean (SD)	Range
**Age**	42(13.428)	45(13.558)	20–69
**Gender**			0–1
Male	523(49.48)	459(46.74)	Dummy (1)
Female	534(50.52)	523(53.26)	Dummy (0)
**Marital status**			0–1
Married	773(73.13)	621(63.24)	Dummy (1)
Others (single, divorced, widowed)	284(26.87)	361(36.76)	Dummy (0)
**Education**			1–5
Lower secondary	320(30.27)	515(52.44)	1
Upper secondary	465(44.99)	177(18.02)	2
Short-cycle tertiary	149(14.10)	50(5.09)	3
Bachelor’s	120(11.35)	208(21.18)	4
Master’s or doctoral	3(0.28)	32(3.26)	5
**Residence area**			
Rural area	228(21.57)	641(65.27)	Dummy (0)
Urban area	829(78.43)	341(34.73)	Dummy (1)
**Employment**			0–1
Working	691(65.37)	815(82.99)	Dummy (0)
Not working	366(34.63)	167(17.01)	Dummy (1)
	**Mean (SD)**	**Mean (SD)**	
**Happiness**	6.961(2.029)	7.651(1.700)	0–10
**Life satisfaction**	6.765(2.058)	7.572(1.759)	0–10
**Structural social capital**			
**Interaction with**			
I1: Family and relatives	3.260(1.062)	4.292(0.984)	1–5
I2: Friends	3.353(1.063)	4.233(0.987)	1–5
I3: Neighbors	3.126(1.218)	3.490(1.061)	1–5
I4: Ratio of interaction with Neighbors	3.237(1.248)	3.790(1.199)	1–5
**Cognitive social capital Trust**			
T1: Family and relatives	3.980(0.839)	4.453(0.820)	1–5
T2: Friends	3.416(0.822)	3.366(1.026)	1–5
T3: Neighbors	3.203(0.872)	3.415(0.985)	1–5
**Festival participation**	2.944(1.368)	3.627(1.237)	1–5
	***n* (%)**	***n* (%)**	
(1) I never attend	209(19.77%)	61(6.21%)	
(2) I don’t usually attend	181(17.12%)	120(12.22%)	
(3) I sometimes participate	338(31.98%)	276(28.11%)	
(4) I try to participate every time	118(11.16%)	192(19.55%)	
(5) I usually participate	211(19.96%)	333(33.91%)	

**Table 2:** Pearson correlation matrix.

	(1)	(2)	(3)	(4)	(5)	(6)	(7)	(8)
(1) Interaction with family and relatives	1.000							
(2) Interaction with friends	0.548 ***	1.000						
(3) Interaction with neighbors	0.246 ***	0.217 ***	1.000					
(4) Ratio of interaction with neighbors	0.268 ***	0.243 ***	0.651 ***	1.000				
(5) Trust family and relatives	0.275 ***	0.204 ***	0.128 ***	0.191 ***	1.000			
(6) Trust friends	0.031	0.094 ***	0.065 *	0.054 *	0.282 ***	1.000		
(7) Trust neighbors	0.156 ***	0.175 ***	0.232 ***	0.260 ***	0.364 ***	0.520 ***	1.000	
(8) Festival participation	0.236 ***	0.265 ***	0.241 ***	0.312 ***	0.135 ***	0.103 ***	0.147 ***	1.000

Note: * *p* < 0.05; ** *p* < 0.01; *** *p* < 0.001.

**Table 3:** Information of X axis and Y axis in (a) and (b) of Figure 1.

The Philippines: Name of regions: 1–17, (rank of happiness, rank of overall life satisfaction)			
1. Pangasinan (10, 10)	2. Isabela (7, 9)	3. Bulacan (13, 17)	4. Cavite (1, 2)
5. Mindoro (16, 16)	6. Camarines Sur (3, 8)	7. Iloilo (9, 7)	8. Negros Occidental (14, 15)
9. Cebu (12, 14)	10. Leyte (17, 6)	11. Zamboanga del Sur (2, 5)	12. Misamis Oriental (4, 1)
13. Davao del Sur (5, 3)	14. South Cotabato (6, 13)	15. Agusan del Norte (15, 12)	16. Lanao del Sur (11, 4)
17. Metro Manila (8, 11)			
**Thailand: Name of regions: 18–28, rank (rank of Happiness, rank of overall life satisfaction)**			
18. Bangkok (11, 11)	19. Nontaburi (8, 7)	20. Ayutthaya (6, 6)	21. Chonburi (5, 9)
22. Ubonratchathani(3,3)	23. Nakornpanom (1, 1)	24. Chiang Mai (10, 10)	25. Pitsanuloke (7, 5)
26. Kanjanaburi (9, 8)	27. Nakorn Sri Thammarat (2, 4)	28. Pattani (4, 2)	

**Table 4:** Festival participation and the determinants.

		Model 1			Model 2		
Variable		Coef.	Odds	z	Coef.	Odds	z
Age	Age	−0.0035	0.997	−1.09	−0.0037	0.996	−1.18
Age^2^	Age^2^	0.0001	1.000	0.40	0.0005	1.000	0.20
Gender	Female (ref.)						
	Male	0.0116	1.012	0.14	0.0316	1.032	0.39
Marital status	Other (ref.)						
	Married	0.187 *	1.206	2.04	0.176 *	1.193	1.92
Education	Education	−0.0985 **	0.906	−2.64	−0.092 **	0.912	−2.47
Working	Working	0.123	1.131	1.24	0.118	1.125	1.19
Urban	Rural (ref.)						
	Urban	−0.449 ***	0.638	−4.92	−0.452 ***	0.636	−4.96
Country	Thai (ref.)						
	Filipino	−0.734 ***	0.480	−7.94	−0.440 ***	0.643	−4.33
Trust_family					0.111 *	1.117	2.24
Interaction_FR					0.241 ***	1.273	5.97
		cut1: −2.570 (0.172)			cut1: −1.074 (0.302)		
		cut2: −1.595 (0.165)			cut2: −0.084 (0.300)		
		cut3: −0.239 (0.161)			cut3: 1.239 (0.302)		
		cut4: 0.494 (0.162)			cut4: 2.043 (0.304)		
		Log-likelihood = −3065.7718			Log-likelihood = −3040.0829		
		Prob > chi^2^ = 0.000			Prob > chi^2^ = 0.000		
		LR Chi^2^(8) = 186.37			LR Chi^2^(10) =232.94		
		Pseudo R^2^ = 0.030			Pseudo R^2^ = 0.0369		

Note 1: * *p* < 0.05; ** *p* < 0.01; *** *p* < 0.001, std. errors in parentheses. Note 2: Age^2^ means age squared.

**Table 5:** Happiness and the determinants.

		Model 3			Model 4		
Variable		Coef.	Odds	z	Coef.	Odds	z
Age	Age	0.001	0.33	0.33	0.002	1.002	0.54
Age^2^	Age^2^	0.001 *	2.09	2.09	0.001 *	1.001	2.14
Gender	Female (ref.)						
	Male	−0.222 *	−2.41	−2.41	−0.213 *	0.808	−2.29
Marital status	Other (ref.)						
	Married	0.334 **	3.22	3.22	0.329 **	1.390	3.16
Education	Education	0.247 ***	5.6	5.6	0.262 ***	1.299	5.86
Working	Working	0.254*	2.29	2.29	0.257 *	1.293	2.31
urban	Rural (ref.)						
	Urban	−0.147	−1.41	−1.41	−0.120	0.887	−1.14
Country	Thai (ref.)						
	Filipino	−0.204	−1.8	−1.8	1.359 *	3.894	2.38
Festival		0.117 **	3.34	3.34	0.187 **	1.205	3.4
Trust_family		0.123 *	2.26	2.26	0.241 **	1.273	3.04
Interaction_FR		0.255 ***	5.68	5.68	0.295 ***	1.342	4.34
Festival * Country					−0.112	0.894	−1.57
Trust_family * Country					−0.217 *	0.805	−1.98
Interaction_FR * Country					−0.076	0.927	−0.84
		cut1: −0.523 (0.355)			cut1: 0.460 (0.499)		
		cut2: 2.580 (0.354)			cut2: 3.565 (0.500)		
		Log-likelihood = −1650.3327			Log-likelihood = −1646.149		
		Prob > chi^2^ = 0.000			Prob > chi^2^ = 0.000		
		LR Chi^2^(11) = 167.19			LR Chi^2^(14) = 175.56		
		Pseudo R^2^ = 0.0482			Pseudo R^2^ = 0.0506		

Note 1: * *p* < 0.05; ** *p* < 0.01; *** *p* < 0.001; std. errors in parentheses. Note 2: Age^2^ means age squared.

**Table 6:** Overall life satisfaction and the determinants.

		Model 5			Model 6		
Variable		Coef.	Odds	z	Coef.	Odds	z
Age	Age	0.001	1.001	0.28	0.002	1.002	0.68
Age^2^	Age^2^	0.001 **	1.001	2.70	0.001 **	1.001	2.79
Gender	Female (ref.)						
	Male	−0.252 **	0.778	−2.77	−0.238 **	0.789	−2.60
Marital status	Other (ref.)						
	Married	0.338 **	1.402	3.29	0.321 **	1.379	3.11
Education	Education	0.206 ***	1.228	4.79	0.239 ***	1.270	5.46
Working	Working	0.176	1.192	1.60	0.187	1.205	1.69
Urban	Rural (ref.)						
	Urban	0.002	1.002	0.02	0.055	1.056	0.53
Country	Thai (ref.)						
	Filipino	−0.439 ***	0.645	−3.90	1.787 **	5.973	3.15
Festival		0.033	1.034	0.97	0.201 ***	1.222	3.69
Trust_family		0.148 **	1.159	2.73	0.229 **	1.257	2.89
Interaction_FR		0.255 ***	1.290	5.70	0.361 ***	1.435	5.32
Festival * Country					−0.281 ***	0.755	−3.97
Trust_family * Country					−0.132	0.876	−1.22
Interaction_FR * Country					−0.201 *	0.818	−2.22
		cut1: −0.394 (0.346)			cut1: 1.096 (0.496)		
		cut2: 2.383 (0.349)			cut2: 3.888 (0.500)		
		Log-likelihood = −1756.7581			Log-likelihood = −1743.6072		
		Prob > chi^2^ = 0.000			Prob > chi_2_ = 0.000		
		LR Chi^2^(11) = 173.04			LR Chi^2^(14) = 199.34		
		Pseudo R^2^ = 0.0469			Pseudo R^2^ = 0.0541		

Note 1: * *p* < 0.05; ** *p* < 0.01; *** *p* < 0.001; std. errors in parentheses. Note 2: Age^2^ means age squared.

**Table 7:** Direct, indirect, and total effects for the relationships between social capital and subjective wellbeing mediated by festival participation.

	Happiness		Life Satisfaction	
Social Capital	Trust Family/Relatives	Interaction with Family/Relatives	Trust Family/Relatives	Interaction with Family/Relatives
*n*	2035	2035	2035	2035
Total effect	0.1898 *** (0.0535)	0.2916 *** (0.0439)	0.2034 *** (0.0530)	0.2777 *** (0.0436)
Direct effect	0.1744 ** (0.0536)	0.2703 *** (0.0442)	0.1968 *** (0.0531)	0.2711 *** (0.0440)
Indirect effect	0.0155 * (0.0061)	0.0214 ** (0.0070)	0.0066 (0.0042)	0.0066 (0.0062)

Note: * *p* < 0.05; ** *p* < 0.01; *** *p* < 0.001; std. errors in parentheses.

## Data Availability

All important information and the dataset are presented in acknowledgements.
